# IgG and IgM differentiation in a particle-based agglutination assay by control over antigen surface density

**DOI:** 10.1063/5.0196224

**Published:** 2024-06-13

**Authors:** Shanil Gandhi, Xhorxhina Shaulli, Jeppe Fock, Frank Scheffold, Rodolphe Marie

**Affiliations:** 1 Department of Health Technology, Technical University of Denmark, 2800 Kongens Lyngby, Denmark; 2 Department of Physics, University of Fribourg, Chemin du Musée 3, CH-1700 Fribourg, Switzerland; 3 Scalevate I/S, 2640 Hedehusene, Denmark

## Abstract

Point-of-care (POC) testing offers fast and on-site diagnostics and can be crucial against many infectious diseases and in screening. One remaining challenge in serological POC testing is the quantification of immunoglobulin G (IgG) and immunoglobulin M (IgM). Quantification of IgG/IgM can be important to evaluate immunity and to discriminate recent infections from past infections and primary infections from secondary infections. POC tests such as lateral flow immunoassays allow IgG and IgM differentiation; however, a remaining limitation is their incapacity to provide quantitative results. In this work, we show how samples containing IgG or IgM can be distinguished in a nanoparticle-based agglutination biosensing assay by tuning the density of antigens on the nanoparticles' surface. We employ direct STochastic Optical Reconstruction Microscopy to quantify the accessible SARS-CoV-2 trimeric spike proteins conjugated to magnetic nanoparticles at a single-particle level and gain insight into the protein distribution provided by the conjugation procedure. Furthermore, we measure the anti-SARS-CoV-2 IgG/IgM induced agglutination using an optomagnetic readout principle. We show that particles with high antigen density have a relatively higher sensitivity toward IgM compared to IgG, whereas low antigen density provides a relatively higher sensitivity to IgG. The finding paves the way for its implementation for other agglutination-based serology tests, allowing for more accurate disease diagnosis.

## I. INTRODUCTION

In the fight against the COVID-19 pandemic, serology did not play the crucial role that it had the potential for. Despite helping to establish links between clusters of COVID-19 outbreaks,^1,2^ factors such as the lack of standardization and knowledge of thresholds of immunity limited the use of serology.^3,4^ Deploying serological tests has been common with the aim of monitoring the spread of COVID-19 infections. Serological assays complement acute assays such as PCR and antigen tests. However, the purpose of the serological test is fundamentally different from the PCR/antigen test,^5^ as it provides valuable insight for vaccine monitoring, detecting prior exposure, and evaluating asymptomatic infections.^4,6^ Traditional laboratory-based serological assays provide valuable insight into immune responses induced by natural infection or vaccination. Still, their implementation as point-of-care (POC) tests faces challenges due to cost, time constraints, and standardization. The polyclonal nature of patient samples makes standardization increasingly difficult, due to the uncertainties about the antigen to be used and which antibody classes to measure (IgG, IgM, IgA, and their combinations).^7,8^ Despite these constraints, large-scale antibody testing can effectively identify the true extent of the pandemic,^9^ pinpointing disease hotspots and high-risk populations for improved isolation and contact tracing in the absence of a vaccine.^10^ Integrating serology testing along with PCR testing into the broader public health strategy can enhance our ability to navigate the complexities of viral spreading.^11^

Currently, serology testing in POC settings for the detection of IgG/IgM is done using lateral flow immunoassay (LFIA). These rapid tests have demonstrated exceptional performance, showcasing their simplicity, cost-effectiveness, speed, and suitability for decentralized testing. They excel in facilitating quick identification, mass screening, and effective containment.^12,13^ However, LFIA is limited in sensitivity and fails to provide a quantitative assessment of the detected analyte, which is essential for testing the efficiency of vaccines against new variants and correlating antibody levels to immunity against COVID-19 infection.^14–16^ Enzyme-linked immunosorbent assay (ELISA) serves as a compelling alternative to LFIA, offering enhanced quantitative diagnostic capabilities characterized by superior sensitivity and a broader dynamic range.^17,18^ However, ELISA demands a longer time for result generation, specialized training, and laboratory expertise for proficient execution, and lacks decentralization.

In this study, we utilize an automated real-time POC test based on a particle-based immunomagnetic assay (IMA). IMA uses a pulsed magnetic field to form magnetic chains of magnetic nanoparticles (MNPs), and subsequently the agglutination of MNPs is quantified by measuring the modulated transmitted light in response to an alternating magnetic field.^19–21^ This optomagnetic (OM) readout utilizes the correlated optical and magnetic anisotropy of MNP chains; The MNP chains align in the magnetic field direction due to the magnetic anisotropy, whereas the optical anisotropy results in a MNP chain orientation dependent transmission of light. When the MNPs form aggregates, both the magnetic and optical anisotropy increase. The method does not provide a direct measure of the change in magnetic anisotropy, but rather a combination of the change in magnetic and optical anisotropy.^22,23^ The combined optical and magnetic effects have been shown to generate a larger signal for particle agglutination compared to the pure magnetic signal.^24^ In contrast to recent work where quantification of MNPs was performed in the absence of a magnetic field,^25^ IMA is performed in solution and does not require any magnetic surfaces.

An IMA-based total SARS-CoV-2 antibody assay has previously been commercialized by BluSense Diagnostics APS and showed good correlations against chemiluminescence immunoassay (CLIA) and ELISA.^26,27^ In the presence of SARS-CoV-2 antibody (IgG or IgM), the protein MNP conjugates undergo agglutination, resulting in the change of optical and magnetic anisotropy.

**FIG. 1.**
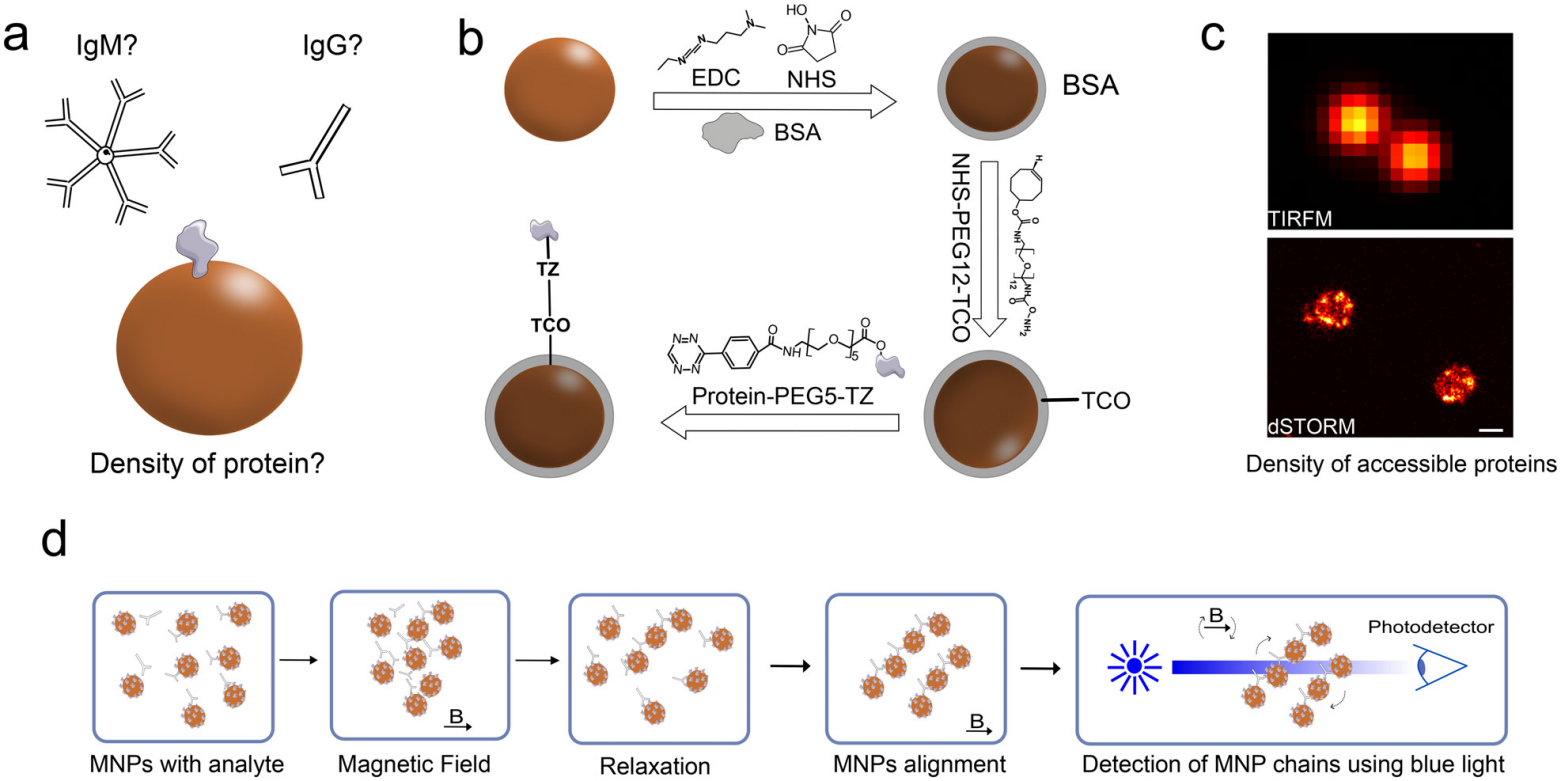
Assessing the structure–function relationship for protein-conjugated MNPs. (a) We aim to establish if varying the density of protein per MNP can influence the detection of antibodies. (b) MNPs were conjugated using TCO-TZ click chemistry on BSA-passivated MNPs. (c) Conjugated nanoparticles were characterized for accessible protein using dSTORM to determine the density of active sites. Top image: diffraction limited TIRF image. Bottom image: reconstructed dSTORM image (scale bar 100 nm). (d) The conjugates were further tested with an immunomagnetic assay on a commercial BluSense instrument. MNPs functionalized with COVID antigen are allowed to react with the COVID antibody present in the biological media. Agglutination of MNP causes antibodies to bind to MNPs under the influence of a magnetic field, thus forming chains. MNP chains repeatedly align and relax in an oscillating magnetic field modulating the transmittance of light measured using the lock-in technique (optomagnetic signal).

Here, we modify the assay to develop a method to differentiate between IgG and IgM samples. MNPs used in the assay are conjugated with the SARS-CoV-2 trimeric spike protein at different protein coverages [Figs. 1(a) and 1(b)]. The conjugates protein density was assessed using a bicinchoninic acid (BCA) assay and direct stochastic reconstruction microscopy (dSTORM),^28^ a single-molecule localization microscopy (SMLM) technique, is used to assess the accessibility of spike proteins. SMLM allows the characterization of nanoparticles at the single-molecule level^29^ [Fig. 1(c)]. With single-molecule resolution, we aim to investigate particle-to-particle variations and the heterogeneity within the protein coating. dSTORM involves capturing images of anti-spike antibodies tagged with a red-emitting synthetic dye Alexa647 and bound to the conjugates. Through the use of elevated laser power to excite the dye molecules in a cysteamine-enriched buffer, we induce controlled fluorescence intermittency, or “blinking,” of the dye^30,31^ to image the accessible spike proteins for each conjugate density.

We observed that higher spike proteins per MNP enhance IgM detection, whereas, in contrast, lower spike protein density increases the detection of IgG [Fig. 1(d)]. We propose a mechanistic model explaining this difference: a high-density spike protein allows both arms of the IgG molecule to bind to the same particle, thus hindering cross binding and agglutination of particles, whereas IgM, with its pentameric structure, is still able to cross-link particles and induce agglutination.

## II. RESULTS

### A. Conjugation of magnetic nanoparticles (MNPs)

The MNPs were conjugated with the antigen using a bio-orthogonal reaction where a cocktail of streptavidin and trimeric spike protein modified with s-tetrazine (Tz) was used at a fixed ratio (95% spike:5% streptavidin). The MNPs were first functionalized with BSA and then trans-cyclooctene (TCO) derivatives^32^ [Fig. 1(b)]. DLS measurements on the conjugates confirmed stability of the particle suspension and the expected change in the hydrodynamic size (Fig. S1). Moreover, a BCA assay showed that the density of spike proteins conjugated to the MNPs was 30% lower than expected from the reaction stoichiometry (Table I). This confirms that the concomitant conjugation with the antigen and streptavidin leads to an efficient tuning of the density of spike proteins on the MNPs. Also, streptavidin allowed for MNPs to be immobilized on a surface for dSTORM imaging.

**TABLE I.** Quantitative analysis of the number of proteins per particle. The MNPs were conjugated with a known quantity of streptavidin and spike protein at a fixed 1:19 ratio to reach an expected area per protein. The MNP conjugates were characterized for their density using a BCA assay by determining the amount of spike protein left in the supernatant after conjugation. Based on the average surface area of the MNP, the number of spike proteins conjugated to MNPs was estimated.

Expected area (nm^2^) per protein	Measured area (nm^2^) per protein	Proteins per MNP
150	195	382
300	373	199
700	887	83
1400	2367	31

The high protein conjugation density corresponding to a target of 150 nm^2^ per spike protein was designed to ensure that the distance between two spike proteins on the MNPs is similar to the distance between two antigen-binding domains (Fab) of IgGs, typically around 13–14 nm.^33,34^ The slightly lower density obtained using the BCA assay corresponds to a mean distance between antigens around 14 nm, thus still a distance similar to the space between the IgGs' two binding epitopes. Conversely, the conjugate with 300 nm^2^ per spike protein (D300) results in a distance between spike proteins slightly greater than the distance between two Fab domains of IgGs. For the low-density conjugates, 700 nm^2^ (D700) and 1400 nm^2^ (D1400) per protein, the spacing between two spike proteins is expected to be more than two times the distance between two Fab domains on IgG.

The conjugation density estimated from the BCA assay provides a bulk measurement for each conjugate. To assess the heterogeneity arising from the conjugation procedure at a single-particle level and to determine the number of accessible and active Trispike proteins for each conjugate density, we used super-resolution microscopy.

### B. Characterization of MNP-conjugated structure

**FIG. 2.**
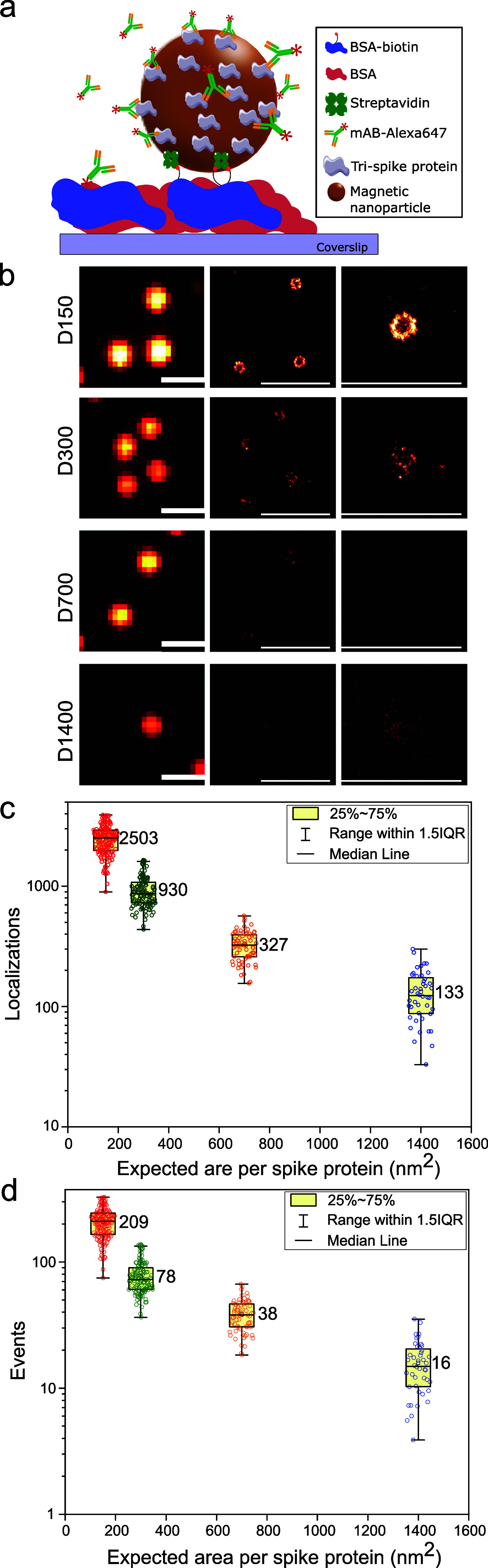
Characterization of MNP conjugates using dSTORM super-resolution microscopy. (a) MNP conjugates were immobilized on a BSA/BSA-biotin coated glass coverslip. (b) Images of D150, D300, D700, and D1400 conjugates in TIRF (left) and reconstructed dSTORM images showing densities of localizations (scale bar is 1000 nm). (c) The number of localizations per particle recorded during the length of the dSTORM movie. (d) The number of bound antibodies calculated using calibration. Dots represent individual MNP conjugates.

The conjugates were immobilized onto a glass coverslip coated with BSA/BSA-biotin for subsequent dSTORM imaging [Fig. 2(a)]. Alexa647-labeled anti-spike antibodies were bound to the MNPs and imaged using dSTORM under total internal reflection (TIR) illumination. Individual MNPs appear as individual dark spots in the bright field images [Fig. S3(a)] and bright spots in the diffraction limited images [Fig. 2(b) and Fig. S3(b)]. However, using the blinking of the fluorophore, we can localize blinking events in each frame and reconstruct a super-resolution image of each particle [Fig. 2(b)]. The reconstructed single-particle dSTORM image of a particle represents the density of detected localizations and is a measure of the accessibility of binding sites on MNP conjugates.

The number of localizations scales with the density of spike proteins [Fig. 2(c)]. However, the absolute number of localizations is not a direct measure of the number of bound antibodies, as it depends on the blinking kinetics and the imaging conditions, i.e., the illumination power and the length of the movie. To estimate the number of bound antibodies from the number of localizations, we followed Woythe *et al.*^35^ and calibrated the mean number of localizations per labeled monoclonal antibody (mAB) at the illumination power used for imaging the high and low-density conjugates [Figs. S4(a) and S4(b)]. We then show the number of “events” in Fig. 2(d) corresponding to the estimated number of labeled antibodies bound to accessible spike proteins per particle. The number of bound antibodies quantified by dSTORM scales linearly with the density of spike proteins conjugated to MNPs, showcasing the effective functionalization of MNPs with a controlled density of spike proteins. The absolute number is about 50% lower than the total number of spike proteins measured by a BCA assay. The measured surface area (nm^2^) per mAB is shown in Table II.

**TABLE II.** Correlation between spike protein per MNP from the BCA assay and antibody bound per MNP from dSTORM. Measured area (nm^2^) per antibody obtained from the number of “events” from dSTORM is compared to the measured area (nm^2^) per spike protein obtained from the BCA assay.

Expected area per protein (nm^2^)	Measured area per protein (nm^2^)	Measured area per mAB (nm^2^)
150	195	356
300	373	955
700	887	1960
1400	2367	4656

### C. Functionality of MNP conjugates

**FIG. 3.**
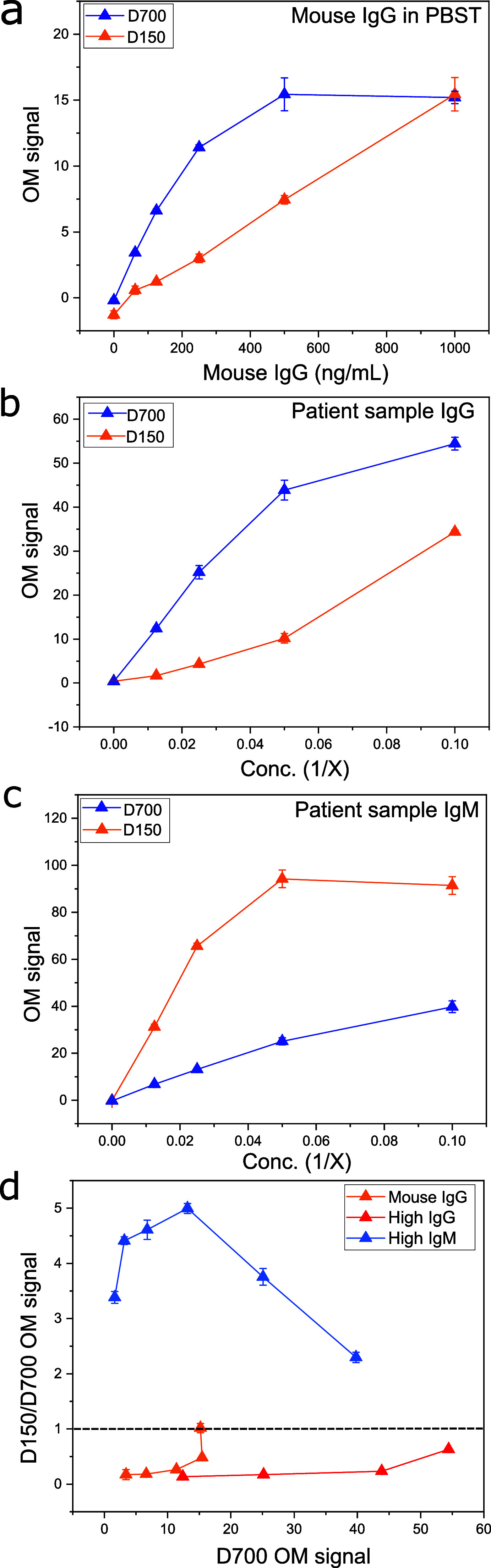
Performances of MNPs in an optomagnetic assay. (a) Dose–response of high (D150) and low (D700) conjugates with mAb IgG in PBST. (b) Dose–response curve with a dilution series of patient sample high in IgG in buffer (20 mM Tris, 150 mM NaCl, 0.05% Tween, and pH 8). (c) Dose–response curve with a dilution series of a patient sample infected with COVID-19 containing IgM. (d) D150/D700 ratio of the OM signal obtained with high-(D150) and low-density conjugate (D700) for Mouse IgG, high IgG, IgM samples, and patient samples. The x-axis shows the OM signal for D700.

The functionality of the MNP conjugates was tested with an immunomagnetic assay. First, we generated a dose–response curve using a serial dilution of anti-spike mouse IgG antibody to understand the functionality of the conjugates. Results from D300 and D1400 conjugates can be found in Figs. S7 and S8, respectively. Figure 3(a) shows the dose–response OM signal for a lower density conjugate (D700), which saturates at 500 ng/ml and the higher density conjugate (D150). Below saturation, the OM signal of the conjugate D700 is higher than D150. A dose–response performed on MNPs conjugated with 5% streptavidin, without spike proteins, showed no unspecific binding of mouse IgG under buffer conditions (Fig. S7).

We further tested the responses of the conjugates with a polyclonal IgG patient sample obtained by pooling patient samples that were infected or vaccinated, ensuring that the sample contained mostly IgG [Fig. 3(b)]. The polyclonal sample also shows the highest signal for the low-density conjugate; however, the signal saturates at a higher value. In addition, we observe a positive curvature for the high-density conjugate.

Next, the conjugates D150 and D700 were tested using a patient sample with high IgM levels determined by the supplier (Boca Biolistics), using the DiaSorin LIAISON^®^ SARS-CoV-2 IgM assay [Fig. 3(c)]. Using the D150 conjugate leads to a higher OM signal than using the D700 conjugate for all dilutions of the sample, although the signal saturates above 20 times the dilution.

In conclusion, the D150 conjugate has the highest response to high IgM samples, while D700 has the highest response in high IgG samples. This appears clearly when plotting the ratio of the D150 signal to the D700 signal against the measured OM signal from D700 [Fig. 3(d)]. A ratio below 1 predominantly indicates the presence of IgG in the sample. Conversely, a ratio above 1 indicates a predominance of IgM in the sample. If either the D150 or the D700 OM signal saturates, the ratio tends to 1, and the assay cannot discriminate between the IgG and IgM responses.

**FIG. 4.**
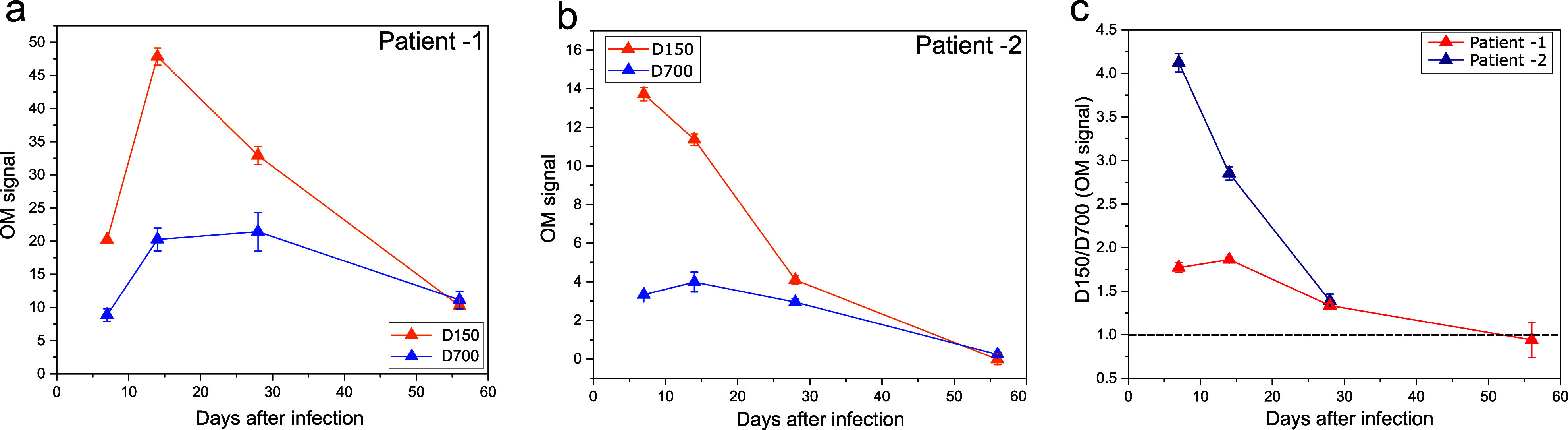
Performance of MNPs against samples taken from COVID-19-infected patients. (a) and (b) The OM signal obtained with D150 and D700 conjugates for two patient sample series collected post-infection. The patient samples were diluted 20× in SDB. (c) The D150/D700 ratio for patient 1 and 2 samples, plotted against the time after infection. The patient 1 sample lacks data points for day 56 because only data points with D150 values above limit of detection are shown.

Finally, we used the comparison of the D150 and D700 responses to test COVID-19-infected patient samples collected from two patients over 8 weeks. We expected that the generation of IgM would be initiated within a few days of the infection,^17^ followed by the onset of IgG and a decrease in IgM. In fact, we detected a higher optomagnetic signal from the D150 conjugate compared to D700 in both patient samples (D150/D700 ratio above 1). The OM signal of both conjugates gradually decreases with time post-infection, and at the last time point of sample collection, eight weeks post-infection, the D150/D700 ratio is 1 [Figs. 4(a) and 4(b)]. Comparing the D150/D700 ratio of the samples of patients 1 and 2 [Fig. 4(c)] shows that the ratio decreases throughout the infection, indicating an increment in IgG over IgM as expected. Patient sample measurements were conducted using 20× dilutions, followed by additional dilutions. Notably, there was no observed saturation of the OM signal throughout these dilution steps.

## III. DISCUSSION

Magnetic nanoparticles were functionalized with Trispike protein at increasing protein densities using a biorthogonal TCO-Tz reaction [Fig. 1(b)]. The reaction fast kinetics and high specificity in aqueous buffer allowed for a quick conjugation reaction between Tz-labeled Trimeric spike protein and TCO functionalized MNPs at physiological conditions.^32^ The protein density was adjusted to achieve a specific range of distance between the spike proteins. For the highest density conjugate (D150), this distance is shorter than the distance between the Fab domains on IgG. This density was then gradually decreased, so the average distance between the spike proteins exceeded the distance between the Fab-binding domains of IgG for the D700 and D1400 conjugates. The protein density of the conjugate estimated from the bulk BCA assay confirms the range of distances between individual spike proteins within 30% of the values expected from reaction stoichiometry.

While the BCA assay measures the average amount of conjugated proteins in bulk, localization microscopy probes the amount of antibody bound to the MNPs, i.e., the accessibility of the spike proteins at the single-particle level. In this context, single-molecule data inform on particle-to-particle variability of bioconjugation. Qualitatively, the reconstructed images displaying the particles as “donut”-shaped density maps are consistent with a homogeneous distribution of proteins on the spherical surface of the MNPs. Quantitatively, the number of mABs detected in the dSTORM images is derived from the number of localizations using a calibration of the mean number of localizations per mAB. The results suggest 50% antigen coverage with the antibody; however, this result is highly dependent on the calibration of the blinking kinetics, which is in turn dependent on the imaging conditions. There are a number of artifacts of the dSTORM imaging itself that could explain this discrepancy. First, particles are deposited on a surface leading to an inaccessible surface (e.g., the bottom surface of particles). Next, the TIR illumination leads to an exponentially decaying illumination of the particle since the diameter coincides with the characteristic decay length.^36^ It is, thus, expected that the blinking dynamics of the fluorophores immobilized directly on the surface of the coverslip and of fluorophores on the surface of the magnetic nanoparticles are different. Finally, scattering, which is particular to the iron core of MNPs, may prevent us from detecting blinking events from the particles' top side facing away from the objective. All three effects potentially lead to a lower total number of mAb calculated from the super-resolution experiment. Moreover, the uncertainty in the determination of the concentration of MNPs will add to the uncertainty in the number of spike proteins per particle determined from the BCA assay. The concentration is estimated from UV-VIS measurements using an extinction coefficient determined from unconjugated particles and the concentration provided by the MNP supplier. This method is not ideal as the particles slightly change the extinction coefficient after conjugation, and the precision of the concentration from the supplier is unknown. We estimate the total systematic error in concentration determination to be 20%–30%.

**FIG. 5.**
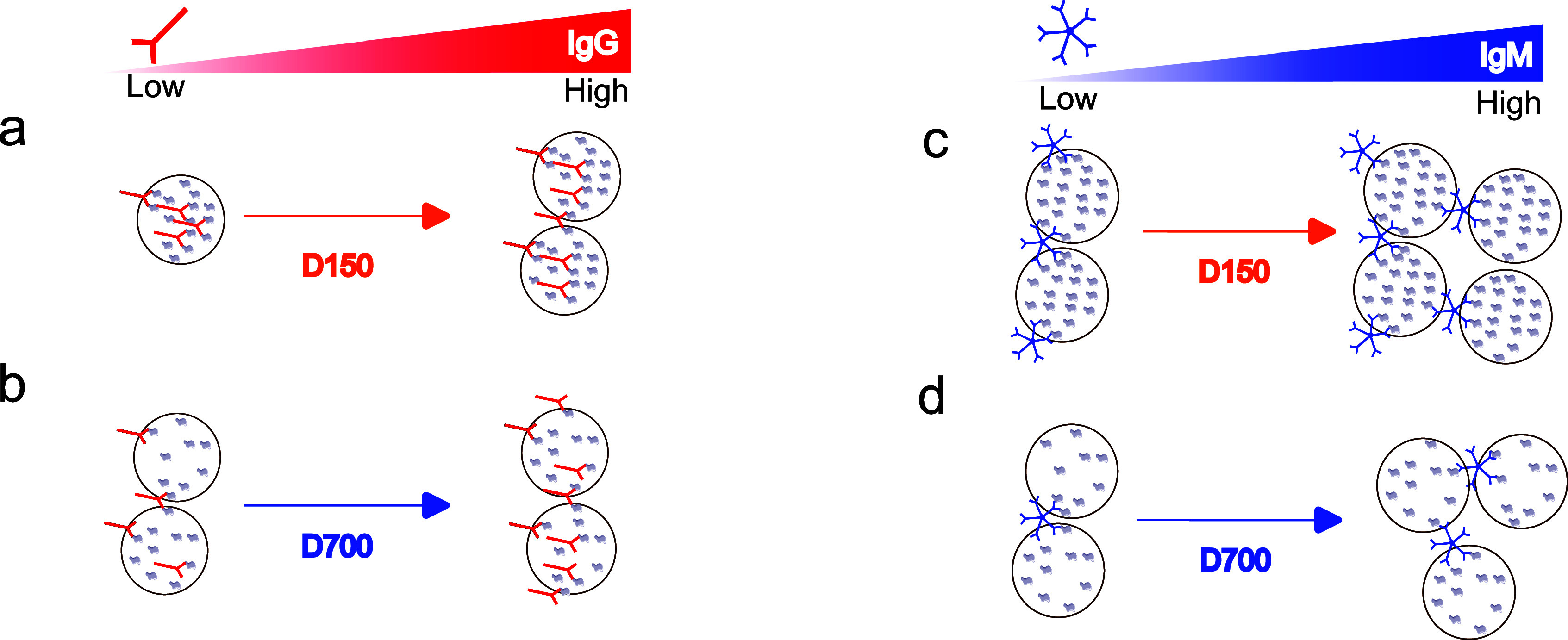
Mechanistic interpretation of the structure–function relationship. IgG and IgM cross-link MNPs differently for different binding site densities. (a) High-density MNPs (D150) at low IgG concentration mainly bind to one MNP, which gradually reduces when IgG concentration increases. (b) Low-density MNPs (D700) for low IgG concentration have free Fab domains that can cross-link to other MNPs, which further increases at high IgG concentration until all proteins on D700 are occupied. (c) and (d) At low IgM concentrations, the high-density conjugate (D150) forms larger aggregates due to the availability of more Fab-binding domains. The low-density conjugate (D700) cross-links to IgM molecules at low concentrations with smaller aggregates due to the availability of fewer binding sites compared to D150. At higher IgM concentrations, the OM signal increases and saturates for both conjugates.

The functionality of the MNP conjugates was assessed in a dose–response assay using a monoclonal mouse IgG and a pooled polyclonal IgG patient sample. The lower density conjugate D700 led to a higher OM signal than the high-density conjugate D150. A high OM signal is obtained when conjugates form chains upon binding with the analyte. The relationship between the OM signal and the spike protein density can, thus, be explained by comparing the distance between the two spike proteins on the MNP and the distance between the Fab domains of mouse IgG. For the high-density conjugate D150, this distance is short enough to allow individual IgG antibodies to frequently bind to two distinct spike proteins on the same MNP [Fig. 5(a)], leading to fewer chain formations. On the contrary, for low-density conjugates (D700), the distance between the spike proteins is increased, and IgG would more frequently bind to spike proteins on different MNPs. Hence, D700 generally leads to a higher OM signal than D150 across the whole IgG concentration range for monoclonal and polyclonal IgG samples [Fig. 5(b)]. At high IgG concentration, the density of the available binding sites on the MNPs decreases, favoring the binding of IgG to spike proteins on different MNPs and the formation of chains. Thus, high- and low-density conjugates lead to the same OM signal [Fig. 5(a)].

The conjugates were then tested with samples with high IgM content [Fig. 3(c)]. The OM signal of D150 was higher than D700 in contrast to the results obtained with the IgG samples. This can be explained by considering that IgM is an antibody with a pentameric structure and can thus bind to two Fab domains on the same particle and still cross-link to a spike protein on a different particle to form MNP chains with Fab-binding domains [Fig. 5(c)]. Hence, the OM signal is directly scaling with the number of binding sites on the MNPs, and hence, high-density conjugates have a higher OM signal [Fig. 5(c)] compared to the low-density conjugate D700 [Fig. 5(d)]. As the IgM concentration increases, the OM signal increases for both conjugates [Figs. 5(c) and 5(d)].

Leveraging the ability of D150 and D700 to discriminate IgG and IgM, the conjugates were tested with COVID-19-infected patient samples collected from the patients over eight weeks (Fig. 4). It is known that an infected individual develops IgM as an immune response during the first 2–3 weeks of infection.^18,37,38^ This is confirmed by the high optomagnetic signal obtained with D150 conjugates. The OM signal decreases over time as the patient gets healthier, with the body maturing the affinity of the antibodies and starts producing IgG.^39^ Moreover, the D150/D700 ratio allows us to evaluate the sample content for all IgG/IgM concentrations and monitor the IgG and IgM levels after infection [Fig. 4(c)]. Also, using the D150/D700 ratio mitigates the influence of patient-to-patient absolute IgG/IgM level variation as long as the OM signal is not allowed to saturate.

Serology testing offers an additional safeguard for confirming SARS-CoV-2 infected patients when the viral load is below PCR tests' limit of detection.^40,41^ The challenge that persists for serology tests is a lack of quantitative detection capabilities to distinguish between IgG and IgM antibodies in a POC setting. We address this by developing a method that exploits the relationship between assay signal and antigen density on the nanoparticles to discriminate IgG or IgM. This approach can be extended to other particle-based agglutination serology assays, such as latex turbidimetric serology tests. Furthermore, for agglutination-based total-antibody serological assays, the control of antigen surface density is expected to be crucial for controlling the reactivity to the different isotypes. Variability of isotype reactivity will pose further challenges for the standardization of serological assays and consequently limit serology testing to meet its full potential.

## IV. SUMMARY AND CONCLUSIONS

In summary, we demonstrate how to discriminate SARS-CoV-2 immunoglobulin IgM and IgG in a particle-based agglutination assay using an automated real-time immunomagnetic assay. We achieved this by controlling the density of the protein conjugated to magnetic nanoparticles using TCO-Tz click chemistry. A BCA assay was used to measure the spike protein density on the magnetic nanoparticles. Furthermore, we used single-molecule localization microscopy to image Alexa647-labeled anti-spike antibodies bound to the MNPs conjugated. Notably, the single-molecule data confirm that the spike protein are effectively diluted on the nanoparticle surface by the conjugation protocol, correlating single-molecule imaging data and chemical characterization data obtained through the BCA assay. This connection highlights that the conjugates' ability to differentiate IgG and IgM within COVID-19 patient samples is, in fact, related to the spike protein density. Future efforts could improve the quantification of single-molecule data by clustering techniques and correcting intrinsic artifacts of single-molecule localization microscopy applied to scattering nanoparticles.

## V. METHODS

### A. Materials

The MNPs, purchased from Ademtech (Carboxyl-Adembeads, Ademtech), are 150 nm in size and have a density of 2 g/cm^3^ with a coating of COOH groups at a density of 400 *μ*mol/g. Chemicals used for functionalization were Tris(hydroxymethyl)aminomethane (Tris), 2-(N-morpholino)ethanesulfonic acid (MES), phosphate-buffered saline (PBS), Tween-20, sodium azide, 1-ethyl-3–(3-dimethylaminopropyl)carbodiimide (EDC), and N-hydroxysuccinimide (NHS), 4–(2-hydroxyethyl-1-piperazineethanesulfonic acid) (HEPES), bovine serum albumin (BSA), dimethyl sulfoxide (DMSO) all purchased from Sigma Aldrich, as well as NHS-PEG12-TCO, NHS-PEG5-TZ, (BroadPharm) and TZ-PEG(5 kDa)-Me (Sigma Aldrich, click chemistry tools). For bioconjugation, we used PD SpinTrap G25 (Cytiva), bicinchoninic acid (BCA) assay kit (Thermo Scientific), Spike S1 protein Mouse anti-SARS-CoV-2, unconjugated, monoclonal antibody (AcroBiosystems), and stabilized Wuhan SARS-CoV-2 trimeric spike protein (ExcellGene). The equipment used for bioconjugation included a magnetic separator (homemade), an ultrasonic homogenizer (sonicators), an ultrasonic bath, a pulsed vortexer (Vortex-Genie Pulse, Scientific Instruments, Inc.), a spectrophotometer, and a 96-well plate reader (Thermo Scientific Multiskan FC 357 Microplate Photometer).

### B. Magnetic nanoparticle conjugation

#### 1. BSA passivation

MNPs were conjugated with BSA using EDC/NHS cross-linking chemistry, which is referred to as “BSA passivation.” In brief, the MNPs were equilibrated in 50 mM MES pH 5.5 using the magnetic separator. The carboxyl groups on the MNPs were activated with 0.25 mg EDC per mg of MNP and 1 mg of NHS per mg of MNP. The carboxyl groups were activated for 15 min and washed twice in PBST, pH 7.2. The MNPs were equilibrated in PBST, pH 7.2, and homogenized (20% amplitude, 1 s ON, 2 s OFF, five cycles). The BSA conjugation of equilibrated MNPs was performed in PBST, 7.2 with 100 mg/ml of BSA solution in PBST, pH 7.2 for 2 h on a pulsed vortexer (1 s ON, 29 s OFF, 2000 RPM). After conjugation the supernatant was collected in a separate Eppendorf tube for BCA analysis. The particles were washed five times in PBST. For the first two cycles, using a magnetic separator, and for the last three cycles of washing, the particles were resuspended using an ultrasonic bath for 5 min. After the last washing cycle, the particles were suspended in PBST with 0.09% sodium azide for storage to a final concentration of 50 mg/ml.

#### 2. Conjugation of MNPs with TCO

The BSA-passivated nanoparticles were resuspended in PBST to a concentration of 10 mg/ml and incubated with NHS-PEG(12)-TCO (100 mM stock) to achieve a target density of 5 TCO molecules per nm^2^. The mixture was incubated on a pulsed vortexer (1 s ON and 29 s OFF) at 2000 RPM for 60 min. Unreacted TCO molecules were quenched for 15 min with 500 mM glycine and 500 mM Tris, pH 8.9. The TCO-conjugated MNPs were washed five times as mentioned above. After the last washing cycle, the TCO-conjugated MNPs are resuspended in PBST, pH 7.2, to a final concentration of 10 mg/ml. The volume of the conjugate was measured, and the concentration of the MNPs was characterized using UV-vis to assess the bead recovery (loss in washing) as described.

#### 3. Labeling of spike protein with Tz linker

The protein was labeled with a Tz linker to click onto the TCO-conjugated MNPs. The target ratio of the Tz linker to proteins was done at 4 mol per mol of streptavidin (MW weight around 50 kDa) and 20 mol per mol of SARS-CoV-2 trimeric spike protein (Mw of 450 kDa). Initially, the NHS-PEG5-Tz linker (10 mM) was diluted in PBST and DMSO to accommodate the ratio of TZ linker to protein, and DMSO is used to increase the solubility of the Tz linker. The diluted linker was incubated with the protein at the mentioned ratios in PBST for 60 min at 28 °C. The reaction was quenched by adding equal volumes of 500 mM glycine and 500 mM Tris for 5 min. The Tz-linked proteins were transferred into an equilibrated PD SpinTrap (as instructed by the manufacturer) to a volume not exceeding 145 *μ*l. After spinning down, the unconjugated Tz linkers were filtered out, while the proteins with linkers were collected in the supernatant. Some Tz-labeled proteins were kept aside for the BCA assay to quantify the labeling yield.

#### 4. TCO-TZ click reaction

The TCO-conjugated MNPs were clicked with Tz-labeled spike proteins at varying target densities: 83 *μ*g of protein per mg of MNP for D150, 41.6 *μ*g of protein per mg of MNP for D300, 17.8 *μ*g of protein per mg of MNP for D700, and 8.9 *μ*g of protein per mg of MNP for D1400. The required amount of Tz-labeled proteins and TCO-conjugated MNPs were added with 10% DMSO to stabilize the PEG linkers. The clicking reaction was performed at 38 °C for 30 min at 1000 RPM on a thermoshaker. The conjugates were immediately spiked with the mTz-PEG-Tz (5 kDa) quencher to quench the Tz groups on the proteins (density of 0.6 mTz molecules per nm^2^). The conjugates were first homogenized (20% amplitude, 1 s ON, 2 s OFF, 5 cycles) upon spiking to increase the surface area for quencher interaction. The quenching reaction was done for 60 min at 38 °C, 1000 RPM on the thermoshaker. The supernatant was collected after the quenching reaction, and the volume was measured for BCA assay. The conjugates undergo three washes in an ultrasonic bath, each lasting 5 min. Following the final wash, the TCO-Tz clicked conjugates were resuspended in PBST (pH 7.2) at a final concentration of 6 mg/ml and stored at 4 °C for later use. For the optomagnetic assay, conjugates were diluted to 0.24 g/L.

#### 5. Characterization of MNP conjugates

The concentration of MNPs after conjugation was estimated by UV-VIS spectrophotometer at 620 nm, using the excitation coefficient of raw MNPs obtained from the supplier. The hydrodynamic size of the MNP conjugates was determined using back-scattering DLS (Nanotrac Flex) measurement by diluting the conjugates 5× in PBST. The conjugation yield of spike protein onto MNPs was determined using a BCA assay (see supplementary material).

### C. Functionality of MNP conjugates

The MNP conjugates were tested for their functionality using a particle-based agglutination assay developed by BluSense Diagnostics. The assay was performed by mixing equal volumes of 0.24 g/L MNP conjugates and diluted samples [Fig. S2(a)]. The resulting mixture was transferred into a cartridge [Fig. S2(b); blue circle] and loaded into the Blubox, developed by BluSense Diagnostics to perform the assay. Each measurement was duplicated on two instruments to eliminate variation in different conditions.

The Blubox [Fig. S2(c)] utilizes a centrifugal microfluidic platform [Fig. S2(b)] to manipulate the sample on single-use cartridges. Centrifugal and capillary forces transfer the MNP and analyte solutions to the detection pool for measurement.^42^

The MNPs were diluted in sample dilution buffer (SDB), consisting of 20 mM Tris, 150 mM NaCl, 0.05% Tween, and pH 8. The sample was either monoclonal antibodies diluted in PBST (0.05% Tween) or a patient sample diluted in SDB. The patient samples were purchased from PATH (patients 1 and 2) and Boca Biolistics (high IgM sample).

### D. Super-resolution microscopy

dSTORM imaging was performed on a Nikon Ti-Eclipse inverted microscope with an EMCCD camera (Andor iXon Ultra 897) and a TIRF arm to achieve highly inclined illumination and limit the fluorescence background. We used a continuous wave red laser (Coherent Genesis MX-STM) at 1 W output power at 639 nm, providing a single-mode TEM00 Gaussian beam, horizontally polarized. The high-power red laser enabled fluorophores in their excited state, through inter-system crossing, to highly occupy the triplet state where they get trapped. The laser was coupled with a single-mode fiber (S405XP, Thorlabs) in the TIRF arm. Excitation light was focused on the back aperture of a high numerical aperture and magnification objective (100× magnification, NA 1.49 and working distance 0.16–0.09 mm). With an extra magnification lens, the final pixel size was 107 nm. The field of view is 128 × 128 pixels in size. An emission filter was placed in the detection path (ET700/50, Chroma). Stochastic blinking was induced at high laser power (∼2 kW/cm^2^). dSTORM movies were acquired with 60 000 frames at 10 ms exposure for high-density conjugates and with 40 000 frames at 30 ms exposure for low-density conjugates. Detailed explanations about sample preparation, image acquisition, and data analysis can be found in the supplementary material.

## SUPPLEMENTARY MATERIAL

See the supplementary material describing the methods and materials of bioconjugation and dSTORM including Figs. S1–S8.

## Data Availability

The data that support the findings of this study are available from the corresponding author upon reasonable request.
